# *Toxoplasma gondii*-Derived Synthetic Peptides Containing B- and T-Cell Epitopes from GRA2 Protein Are Able to Enhance Mice Survival in a Model of Experimental Toxoplasmosis

**DOI:** 10.3389/fcimb.2016.00059

**Published:** 2016-06-01

**Authors:** Luciana M. Bastos, Arlindo G. Macêdo, Murilo V. Silva, Fernanda M. Santiago, Eliezer L. P. Ramos, Fabiana A. A. Santos, Carlos P. Pirovani, Luiz R. Goulart, Tiago W. P. Mineo, José R. Mineo

**Affiliations:** ^1^Laboratório de Imunoparasitologia “Dr. Mário Endsfeldz Camargo”, Instituto de Ciências Biomédicas, Universidade Federal de UberlândiaUberlândia, Brazil; ^2^Laboratório de Nanobiotecnologia, Instituto de Genética e Bioquímica, Universidade Federal de UberlândiaUberlândia, Brazil; ^3^Centro de Biotecnologia e Genética, Universidade Estadual de Santa CruzIlhéus, Brazil

**Keywords:** *Toxoplasma gondii*, GRA2, monoclonal antibody, B- and T-cell epitopes

## Abstract

Toxoplasmosis is a zoonosis distributed all over the world, which the etiologic agent is an intracellular protozoan parasite, *Toxoplasma gondii*. This disease may cause abortions and severe diseases in many warm-blood hosts, including humans, particularly the immunocompromised patients. The parasite specialized secretory organelles, as micronemes, rhoptries and dense granules, are critical for the successful parasitism. The dense granule protein 2 (GRA2) is a parasite immunogenic protein secreted during infections and previous studies have been shown that this parasite component is crucial for the formation of intravacuolar membranous nanotubular network (MNN), as well as for secretion into the vacuole and spatial organization of the parasites within the vacuole. In the present study, we produced a monoclonal antibody to GRA2 (C3C5 mAb, isotype IgG2b), mapped the immunodominant epitope of the protein by phage display and built GRA2 synthetic epitopes to evaluate their ability to protect mice in a model of experimental infection. Our results showed that synthetic peptides for B- and T-cell epitopes are able to improve survival of immunized animals. In contrast with non-immunized animals, the immunized mice with both B- and T-cell epitopes had a better balance of cytokines and demonstrated higher levels of IL-10, IL-4 and IL-17 production, though similar levels of TNF-α and IL-6 were observed. The immunization with both B- and T-cell epitopes resulted in survival rate higher than 85% of the challenged mice. Overall, these results demonstrate that immunization with synthetic epitopes for both B- and T-cells from GRA2 protein can be more effective to protect against infection by *T. gondii*.

## Introduction

Toxoplasmosis is a congenital or acquired infectious disease caused by *Toxoplasma gondii*, an obligate intracellular protozoan (Dubey, 2010). It is an important cause of neonatal disorders and opportunistic infections in animals and humans (Montoya and Liesenfeld, 2004).

*Toxoplasma gondii* has regulated secretory organelles involved in invasion and replication strategies, which include the apical micronemes and rhoptries, as well as the dense granules (Black and Boothroyd, 2000; Franco et al., 2016).

Host cell invasion by *T. gondii* tachyzoites has been described as process involving the sequential secretion of micronemes and rhoptry proteins (Carruthers and Sibley, 1997). Successful intracellular development is based by active invasion process and formation of a new sub-cellular compartment named the parasitophorous vacuole (PV) (Braun et al., 2008). The dense granules (GRA) proteins are secreted into the PV during invasion in the host cell and remain soluble in the PV lumen or associated with PV membrane (PVM) or tubulovesicular network (TVN) of membranous structure within the PV (Zinecker et al., 1998; Nam, 2009).

Several studies have been conducted with GRA proteins and related with host-parasite interaction and immune response (Sibley et al., 1995; Mercier et al., 1998a; Xue et al., 2008; Zhou et al., 2012). GRA2 can be detected during infections in humans and domestic animals and in experimental models, showing its potential immunogenic capability (Murray et al., 1993; Xue et al., 2008). The GRA2 protein can induce a long-term activation of T helper cell-*T. gondii* specific in humans (Prigione et al., 2000). This immunological response to GRA2 may be important to control infection and immunization studies with the native protein have been shown to elicit protection in mice with acute toxoplasmosis (Sharma et al., 1984; Mercier et al., 1998b).

In order to better understand the role of GRA2 in the adaptive immune responses against *T. gondii*, the major aim of the present study was to map B- and T- cell epitopes by phage display and epitope prediction and to test the ability of two selected linear peptides to protect mice in an experimental model of infection by *T. gondii*.

## Materials and methods

### Parasites and soluble tachyzoite antigen (STAg)

Tachyzoites from RH strain of *T. gondii*, as well as the 2F1 parasite clone used for proliferation assay, were obtained as previously described (Mineo et al., 1993; Teo et al., 2007). Parasite suspensions were washed in PBS, added a protease inhibitor cocktail (Complete Ultra tablets, Roche, USA), and processed for lysis by repeated freezing and thawing cycles, followed by three cycles in cell disruptor for 1 min in ice bath. The preparations were centrifuged at 14,000 × *g* for 30 min at 4°C. After discarding the sediment, the protein concentration in supernatant was determined by the Bicinchoninic acid kit (BCA, Sigma, St. Louis, USA) and aliquots were stored as soluble tachyzoite antigen (STAg) at −80°C until use.

### Production of monoclonal antibodies

Freshly harvested *T. gondii* RH strain tachyzoites were treated with acetone solution (30%), at 4°C for 72 h. Parasite suspensions were washed in PBS and used for immunization of Balb/c mice. Hybridoma production was performed as previously described (Cunha-Júnior et al., 2010). Briefly, hybridomas were produced by fusion of splenocytes extracted from immunized mice with SP_2_O/Ag14 murine myeloma cells (1:1) in the presence of polyethyleneglycol (PEG1500; Sigma-Aldrich) and specific secreting hybridomas were selected by ELISA. The hybridoma (C3C5) selected was cloned by limiting dilution, and isotyped by immunoenzymatic assay (IsoQuick™, Sigma-Aldrich).

### Indirect fluorescent antibody test (IFAT)

The indirect immunofluorescence assay was performed for immunolocalization of the protein tagged by C3C5 mAb against *T. gondii* tachyzoites, as previously described (Ferreira Júnior et al., 2012), with minor modifications. Briefly, parasites were treated with 4% formaldehyde, during 30 min, at room temperature, washed in PBS and placed on glass slides, where they were permeabilized by Triton X-100 0.1% for 10 min at room temperature, and incubated with C3C5 mAb. After washing, slides were incubated with rabbit IgG anti-mouse IgG labeled with fluorescein isothiocyanate (FITC; Sigma). Slides were then analyzed by confocal microscope (LM 510 Meta, Zeiss, Germany). Positive and negative serum samples controls were included in each slide and they were obtained from chronically infected and naive Balb/c mice, respectively.

### 1D- and 2D-immunoblot assays

Immunoblot assays were carried out to characterize the protein recognized by C3C5 mAb. In 1D-immunoblot, STAg was separated on 12% SDS-PAGE under non-reducing conditions and electrotransferred to nitrocellulose membranes, which were blocked by 5% skim milk-PBS-T. Nitrocellulose strips were then incubated with C3C5 overnight at 4°C. The secondary antibody, consisting of mouse anti-IgG labeled with peroxidase (Sigma-Aldrich), diluted at 1:2000, was added to strips for 2 h at room temperature. Reactions were revealed with 10 mg of 3,3- diaminobenzidine tetrahydrochloride (DAB, Sigma-Aldrich) in 15 mL of Tris buffered saline (TBS) and 12 μL of 30% hydrogen peroxide (Sigma-Aldrich) and stopped with several washes in distilled water. A 2D-immunoblot assay was also carried out to evaluate the recognition of STAg protein by C3C5 mAb. Briefly, 0.5 mg of STAg dialyzed in ultrapure water was separated by isoelectric focusing (IEF) on 11-cm immobilized pH gradient strips (ReadyStrip^TM^ IPG Strip pH 3–10) overnight at room temperature, according to manufacturer instructions for equipment and reagents (GE Healthcare, Uppsala, Sweden). After IEF, strips were equilibrated and loaded onto precast 12% polyacrylamide gels. Electrophoresis was performed and 2D-gels were stained with Coomassie brilliant blue G-250H (Sigma-Aldrich) or electrotransferred to nitrocellulose membranes. 2D-immunoblot was performed as described above for 1D-immunoblot. Positive reactions were detected by chemiluminescence (ECL, GE Biosciences Amersham, Piscataway, USA). The corresponding spots were excised from the Comassie brilliant blue stained gel and submitted for analysis by mass spectrometry.

### In-gel trypsin digestion and mass spectrometry

The identification of protein by mass spectrometry was performed as described elsewhere (Shevchenko et al., 1996). The spots of interest were selected and excised manually from 2D gels previously stained with Coomassie brilliant blue. Gel pieces were washed with 25 mM ammonium bicarbonate and 50% ACN, dried by vacuum centrifugation and subsequently treated with 5–7 μg/mL of trypsin (Promega, Madison, USA) according to the manufacturer instructions. The resulting tryptic digests were vacuum concentrated, desalted using a preymmetry column C18 (180 μm in inner diameter × 20 mm long, 5 μm particles) and then fractioned by a C18 reverse phase chromatography column (100 μm × 100 mm, 1.7 μm) on the nanoAcquity UPLC (Waters, Mildford, USA), with load flow of 0.6 μL/min by 50 min/run. Each sample was collected for posterior analysis. The peptides were separated in accordance with gradient steps of water/ACN. The separated peptides were ionized in a capillary under voltage of 3000 V (Micromass Q-Tof MicroTM), fragmented in the positive ion mode, with selection of the relative intensity of at least 10 counts, and analyzed the three most intense ions (scan/s) with collision energy varying between 20 and 95 eV, according to mass/charge(m/z) of peptides. The spectra were analyzed by Protein Lynx Global Server (PLGS) 4.2 (Waters) set up to tryptic digestion with one missed cleavage site, error tolerance of 30 ppm and tolerance for error of 0.3 Da mass equal to the peptides were constructed by the *de novo* sequencing method, and searched in the National Center for Biotechnology Information (NCBI) database.

### Peptide selection from phage display library

A random peptide library of seven amino acids (Ph.D-C7C, New England Biolabs, Hitchin, UK) was used for selection of peptides in fusion with the protein III (pIII) of the filamentous phage (M13) capsid specific to monoclonal antibody C3C5. One well of a microtiter plate (96-well Maxisorp plate, Nunc, Sigma-Aldrich) was coated with C3C5 mAb (1 μg/well in NaHCO_3_ 0.1 M, pH 8.6), and incubated overnight 4°C in a humidified chamber. The reaction was blocked with blocking buffer (NaHCO_3_ 0.1 M, pH 8.6, 5 mg/mL BSA) for 1 h under agitation at 4°C and then washed six times with TBST (50 Tris-HCl mM pH 7.5, NaCl 150 mM, water, 0.1% volume/volume of Tween 20). Immediately, 10 μL of the library diluted in 90 μL of TBST were added and kept under agitation for 1 h at room temperature. Non-ligand phages were removed by washing 10 times with TBST (0.1% Tween-20) in the first two selection cycles and with TBST (0.5% Tween-20) in the third subsequent cycle to increase the stringency. Bound phages were eluted in 100 μL of elution buffer (0.2 M Glicine-HCl, pH 2.2, and BSA 1 mg/mL) during 10 min at room temperature and the supernatant were neutralized with 1 M Tris-HCl (pH 9.1). Aliquots of the eluted phages were used for titer determination. Phage titration and amplification were performed in *E. coli* (ER2738 strain) and the selection was repeated for two additional cycles. Resultant clones were individually reamplified in deep-well plates. These clones were properly characterized by sequencing and immunoenzymatic assays. The colonies derived from biopanning titrated plates were isolated and transferred to wells of culture plates (deepwell type), containing culture of ER2738 in early-log phase (OD 600 ~ 0.3) for the extraction of DNA phage. The plate was sealed and incubated at 37°C for 24 h under agitation (250 rpm). To isolate phage of bacteria, plates were centrifuged at 3700 rpm at 20°C for 30 min. Next, supernatant incubated for 10 min with PEG/NaCl (20% polyethylene glycol 8000 and 2.5 M NaCl). The plates were centrifuged at 3700 rpm at 20°C for 40 min to precipitate the phage. The supernatant was discarded and 100 μL of iodide buffer (10 mM Tris-HCl pH 8.0, 1 mM EDTA, and 4 M NaI) were added to precipitate phage. After being shaken vigorously for 40 s and incubation at room temperature, the plates were centrifuged (3700 rpm, 20°C, 10 min) and the supernatant discarded. The phage DNA was washed with ethanol 70% and centrifuged under the same conditions. Finally, the DNA precipitated was diluted in Milli-Q water. The single stranded DNA quality was verified by electrophoretic run on 0.8% agarose gel stained with ethidium bromide solution.

### Analysis and characterization of clones

Selected phages were submitted to DNA sequencing, which was carried out by using the “Big Dye Terminator” sequencing kit (GE Healthcare), and the universal “primer” (96 M13- 5′- HOCCCTCATTAGTTA GCGCGTAACG 3′–GE Healthcare) to amplify the recombinant amino acids region coded by random peptides fused at the pIII of the M13 phage in the automatic capillary sequencer MegaBace 1000 (GE Healthcare). DNA sequences were then submitted to bioinformatics analyses. Amino acid sequences were deduced according to the nucleotide sequences and analyzed using the translate tool from ExPASy—SIB Bioinformatics Resource Portal (http://web.expasy.org/translate/).

### Bioinformatics

The three-dimensional structure predictions of the GRA2 protein were performed with the I-TASSER server (Roy et al., 2010). The similarity of selected peptides with GRA2 *T. gondii* was performed using BLAST search (Basic Local Aligment Search Tool) (http://www.ncbi.nlm.nih.gov/blast), followed by sequence alignment with ClustalW2 software (http://www.ebi.ac.uk/Tools/msa/clustalw2/). The similarities between selected peptides and proteins deposited were performed using the tool BLAST. Predicted epitopes to T cell and B cell for the protein GRA2 were obtained in Immune Epitope Database and Analysis Resource (http://iedb.org/).

### Peptide design and synthesis

Two peptide sequences from GRA2 were designed and chemically synthesized by Peptide 2.0 Inc. (Chantilly, USA). To increase immunogenicity, peptides were coupled to bovine serum albumin (BSA). The peptide Tx1 (ACEPVSQRASCGGGS) was constructed with 15 residues and peptide Tx2 (RASRVAEQLFRKFLKFAGGGS) was constructed with 21 residues, both containing a 4-aa spacer, GGGS. Three glycines (G) and amidation C-terminal disulfide bond between amino acids 2 and 11 were added in Tx1 peptide sequence. Tx2 peptide possessed the sequence of glycine and C-terminal amidation only. The peptides were lyophilized and later solubilized in Dimethylsulfoxide (DMSO-Sigma).

### Immunization procedure

All experiments were carried out with 6–10 week-old female C57BL/6 mice obtained from Department of Biochemistry and Immunology, School of Medicine of Ribeirão Preto, USP, Ribeirão Preto, Brazil. Animals were maintained under standard conditions in the Bioterism Center and Animal Experimentation, Federal University of Uberlandia, Brazil. All procedures were conducted according to institutional guidelines for animal ethics and the study received approval of the Ethics Committee for Animal Experimentation of the Institution (CEUA-UFU), under the protocol # 029/12. Five groups of 10 mice were immunized subcutaneously with the following formulations: 25 μg of STAg (STAg group), Tx1 (Tx1 group), Tx2 (Tx2 group), Tx1 plus Tx2 (Tx1+Tx2 group), or BSA (BSA group) in 100 μL of sterile PBS added to 100 μL Freund's adjuvant (Sigma) (STAg group). Immunizations were carried out at regular intervals of 15 days in three successive inoculations. The first immunization was performed with Freund's complete adjuvant and the two remaining with incomplete Freund's adjuvant. Blood samples were collected at 0, 15, 30, 45 days after immunization (d.a.i) and the sera analyzed for the presence of specific antibodies. Two weeks after the last immunization (45 d.a.i.), seven animals per group were challenged orally with 30 cysts of *T. gondii* (ME49 strain). All surviving animals were euthanized at 30 days after challenge (75 d.a.i.), the brain tissues were collected, sliced longitudinally and stored at −70°C for quantification of parasite burden by real-time PCR.

### Indirect ELISA for detection of antibodies anti-STAg and anti-peptides

The kinetics of IgG produced by the immunized mice was evaluated by an indirect ELISA. The optimal conditions for ELISA were obtained through block titration of the reagents. Briefly, high affinity microtiter plates (Costar Corning Incorporated, Sigma-Aldrich) were coated with STAg (10 μg/mL) and the peptide mixed (Tx1+Tx2) (1 μg/well) in 0.06 M carbonate buffer (pH 9.6) and incubated overnight at 4°C. Plates were washed with PBS-Tween 0.05% (PBS-T) and blocked with PBS-T plus 1% low fat milk for 1 h at room temperature. Serum samples from immunized mice with BSA, STAg and Tx1+Tx2 were added to the wells in duplicate and incubated for 1 h at 37°C. After washing, plates were incubated with anti-mouse IgG antibody labeled with peroxidase, diluted 1:2000 in PBS-T, for 1 h at 37°C. The reactions were revealed by adding 0.01M 2,29-azino-bis(3-ethylbenzthiazoline-6-sulphonic acid) (ABTS, Sigma-Aldrich) and 0.03% H_2_O_2._The optical density (OD) was determined at 405 nm. Four positive quality-controls and four negative controls (serum from non-immunized animals and uninfected) were included in each plate in order to calculate the cut off, which was established as the mean OD values for negative controls plus three standard deviations. A similar protocol was applied to detect IgG subclasses, IgG1 and IgG2a). After washing with primary antibody, plates were incubated with biotinylated secondary antibodies (Caltag Lab. Inc., South San Francisco, USA) anti-IgG1 mouse diluted 1:4000 with PBS-T 1% or anti-IgG 2a mouse diluted 1:2000. After incubation for 1 h at 37°C, the plates were washed again and incubated (50 μL/well) with streptavidin-peroxidase (Sigma-Aldrich) diluted 1:1000 in PBS-1% BSA T. Results were expressed as ELISA index (EI), as previously described (Silva et al., 2002), according to the following formula: EI = OD_sample_/OD_cut−off_, where values of EI > 1.2 were considered positive.

### Cytokine quantification

Cytokine analysis was performed in serum samples from immunized animals and infected with *T. gondii*. The serum samples were collected after 7 days of infection. The dosage of cytokine in these sera was done using the CBA kit (Cytometric Bead Array—BD Biosciences, Franklin Lakes, USA). The reactions were performed according to the manufacturer's instructions and the analysis was performed on the flow cytometer from BD FACS Canto II.

### Detection of parasite burden by quantitative real-time *PCR*

Parasite burden in the brains of mice was determined by quantitative real-time PCR, as previously described (Wahab et al., 2010), using primer pairs (5′-GCTCCTCCAGCC GTCTTG-3′ and the reverse primer 5′-TCCTCA CCCTCGCCTTCAT-3′) targeting the repetitive 529 bp fragment of *T. gondii* genomic DNA, by SYBR green detection system (Invitrogen, San Francisco, USA). DNA extraction was performed from murine brain tissues using the Wizard SV Genomic DNA kit (Promega) following manufacturer's instructions. DNA concentrations were determined by UV spectrophotometry at 260 nm (NanoDrop® Spectrophotometer 1800 ND-1000, NanoDrop Technologies, Inc, Wilmington, USA) and adjusted to 200 ng/μL with sterile DNAse free water. Assays to determine *T. gondii* tachyzoite loads were performed by real-time PCR StepOnePlus™ PCR Systems (Applied Biosystems, Foster City, USA). The reaction mixtures (25 μL) consisted of 1 μL TaqMan PCR master mix (Applied Biosystems), 100 nM probe, and 900 nM (each) primers, forward and reverse, together with the different samples in triplicate. The amplification conditions comprised 50°C for 2 min, initial activation at 95°C for 10 min, and 45 cycles of denaturation at 95°C for 15 s and annealing/extension at 60°C for 1 min. The parasite counts were calculated by interpolation from a standard curve (10^2^–10^−7^ ng) with DNA equivalents extracted from *T. gondii* tachyzoites included in each run.

### Statistical analysis

Statistical analysis was carried out using GraphPad Prism 5.0 (GraphPad Software Inc., San Diego, USA). Differences between groups were analyzed using ANOVA or Kruskal-Wallis test, when appropriate, followed by Bonferroni or Dunn multiple comparison post-tests to examine all possible pairwise comparisons. Student *t*-test was used for comparison between levels of IgG1 and IgG2a isotypes. The Kaplan-Meier method was applied to estimate the survival percentage of mice at each time point after challenge and survival curves were compared using the Log-rank test. Values of *P* < 0.05 were considered statistically significant.

## Results

### Characterization of C3C5 monoclonal antibody

**Figure 1:**
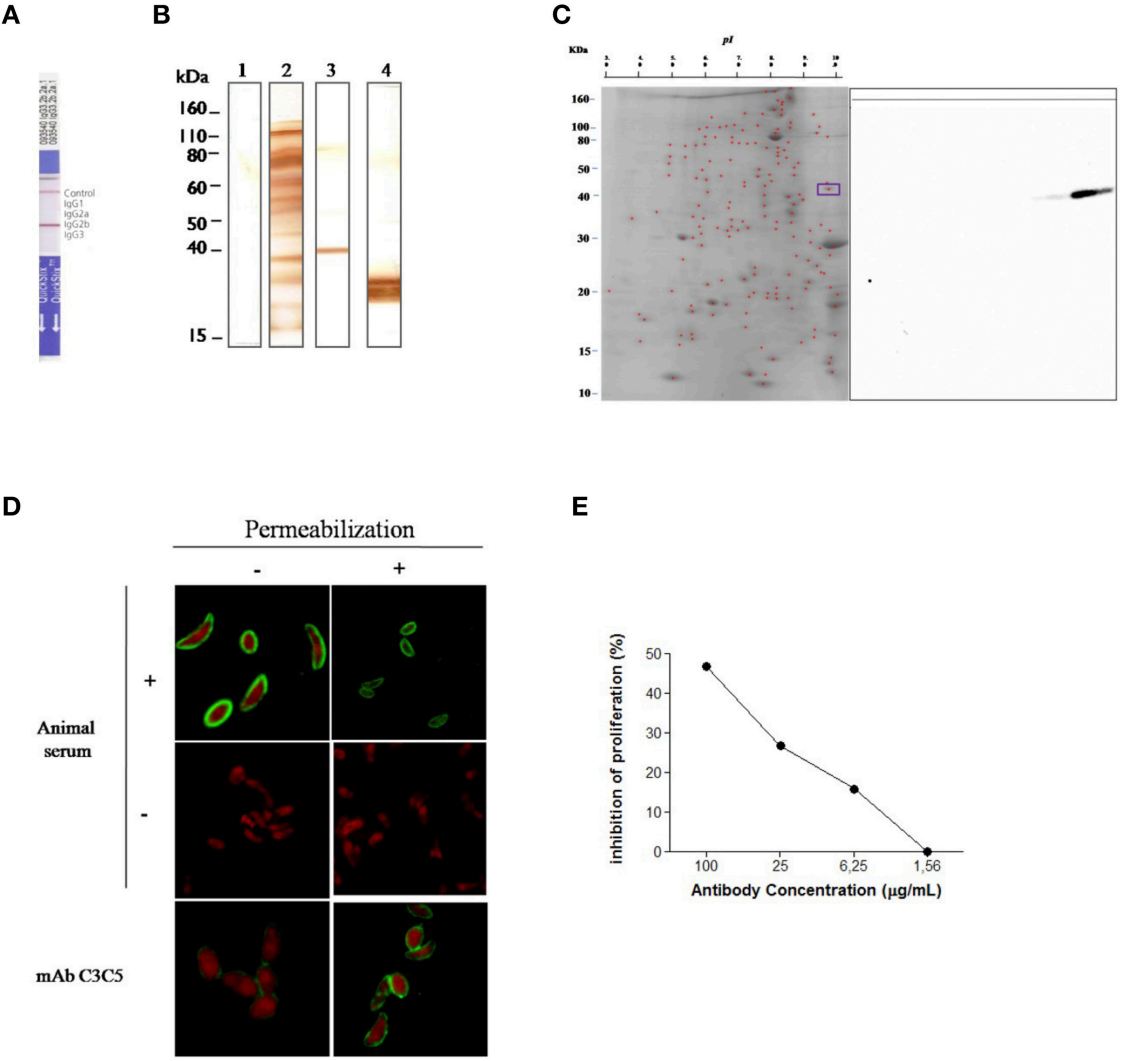
**Selection and immunochemical characterization of monoclonal antibody C3C5 specific for GRA2 protein of ***Toxoplasma gondii***. (A)** Isotyping of C3C5 mAb by using an immunoenzymatic assay in culture supernatant. **(B)** 1D immunoblot of *T. gondii* RH strain tachyzoites solubilized in SDS sample buffer and resolved in 12% SDS-PAGE and probed with no infected mouse serum sample (Lane 1), *T. gondii* infected mouse serum sample (Lane 2), C3C5 mAb (Lane 3), and anti-p30 mAb E9 (Lane 4). **(C)** 2D immunoblot of *T. gondii* RH strain tachyzoites solubilized in isoelectric focalization solution and resolved through pH 3–10 and then in 12% SDS-PAGE. After electrotransference to nitrocellulose membranes, antigens were probed with C3C5 mAb. **(D)** Indirect fluorescent antibody test (IFAT) of *T. gondii* RH strain probed with mice serum no infected (−), infected with *T. gondii* (+) and monoclonal antibody C3C5. Slides containing parasite were permeabilized (+) or not (−) with Triton X-100 0.01% for 10 min. **(E)** Proliferation inhibition of *T. gondii* infection by C3C5 mAb in HeLa. *T. gondii* 2F1 strain tachyzoites were pretreated for 60 min at 37°C, 5% CO_2_ with increased concentration (1.56–100 μg/mL) of C3C5 mAb and irrelevant mouse IgG. The proliferation assay was development from β–gal activity measured at absorbance of 550 nm using a kinetic plate reader (Molecular Devices).

The C3C5 mAb was characterized as belonging to the isotype IgG2b (Figure 1A). To identify the type of epitope recognized by C3C5 mAb, 1D immunoblot was carried out with SDS-soluble antigen in the presence or absence of 2 mercaptoethanol (Figure 1B). The C3C5 mAb recognized a polypeptide of about 45 kDa in non-reducing denaturing condition, whereas this polypeptide could not be visualized when reducing denaturing condition was performed. In contrast, the profile of reactivity for the E9 mAb used as control recognizes an epitope from 30 kDa protein (p30) of *T. gondii* in both conditions (Figure 1B).

To identify the presence of proteins that could be recognized by C3C5 mAb, a 2D immunoblot assay was carried out (Figure 1C). Two spots migrating at 45 kDa were detected, indicating that at least two isoforms of protein contained common epitope recognized by C3C5 mAb. The cellular localization of epitopes in *T. gondii* tachyzoites were examined by immunofluorescence (Figure 1D). Slides incubated with serum from infected animals (positive control), uninfected animal (negative control), and C3C5 mAb were observed by confocal microscopy. C3C5 mAb recognized tachyzoites permeabilized or not, however higher stained can be observed in permeabilized. The proliferation inhibition of *T. gondii* (2F1 strain) was observed by C3C5 mAb (Figure 1E). The highest inhibition profile was obtained with 100 μg/mL of C3C5 mAb, when compared to incubation with the same amount of isotype control.

### Epitope mapping by phage display and peptide synthesis

**Figure 2:**
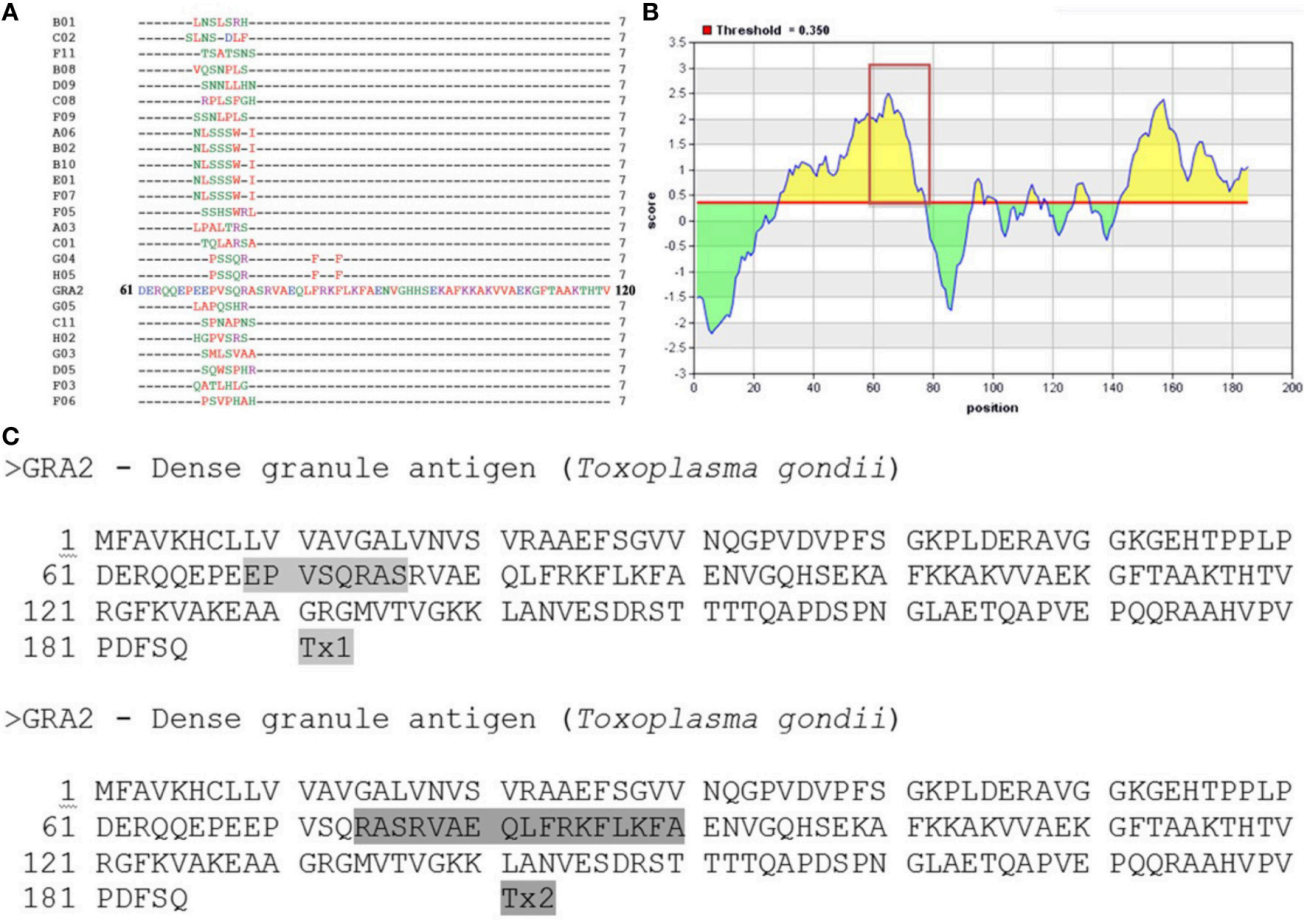
**Epitope mapping of GRA2 by Phage Display and peptides synthesis. (A)** Peptide sequences of 24 phage clones and their consensus sequences, according to the original sequence determined for the C-terminal region of the GRA2 protein. **(B)** Immunodominant region especially for B cell (http://iedb.org/). **(C)** Based on the results of prediction for B cells and T cells into GRA2 structure was designed to be synthesized two peptides, Tx1 and Tx2, respectively.

Twenty-four randomly selected mimotopes were obtained after three rounds of biopanning using a phage displayed 7-mer random peptide library against C3C5 mAb (Figure 2A). Alignment analysis revealed the consensus sequence comprised between amino acids 68–76 of protein GRA2 of *T. gondii* and revealed be an immunodominant region for B cell (highlighted in Figure 2B). *In silico* prediction shows that MHCI/MHCII epitopes can be found within the same region of the B cell epitope. In that sense, we synthesized two peptides, one for B cell (Tx1) and another for T cell (Tx2) to be used in immunization protocols (Figure 2C).

### Protein modeling

**Figure 3:**
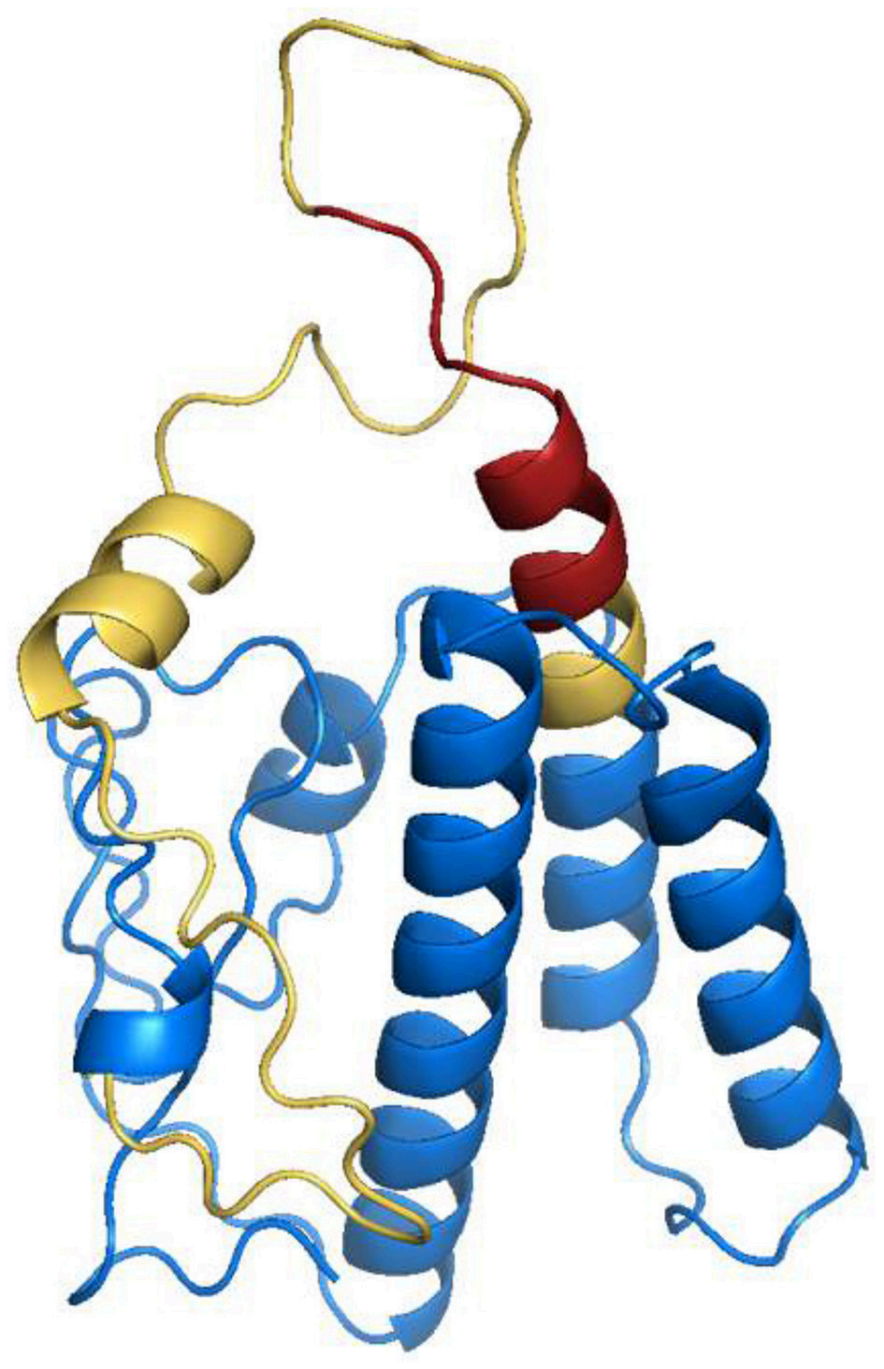
**Structural modeling of dense granule 2 (GRA2) of ***Toxoplasma gondii*****. *In silico* analysis of three-dimensional structure of GRA2 protein were performed to search for possible intrinsically unstructured protein (IUP) domains, evidencing predicted epitopes for B (highlighted in yellow) and T (highlighted in red).

To confirm the surface exposure probability of the consensus amino acids sequence, we have performed a simulation to generate a 3D structure of the GRA2 protein. The putative localization of the epitope within the structure was shown in Figure 3, corroborating with the possible antibody binding region in the external sequences of the predicted protein.

### Survival of immunized and challenged animals

**Figure 4:**
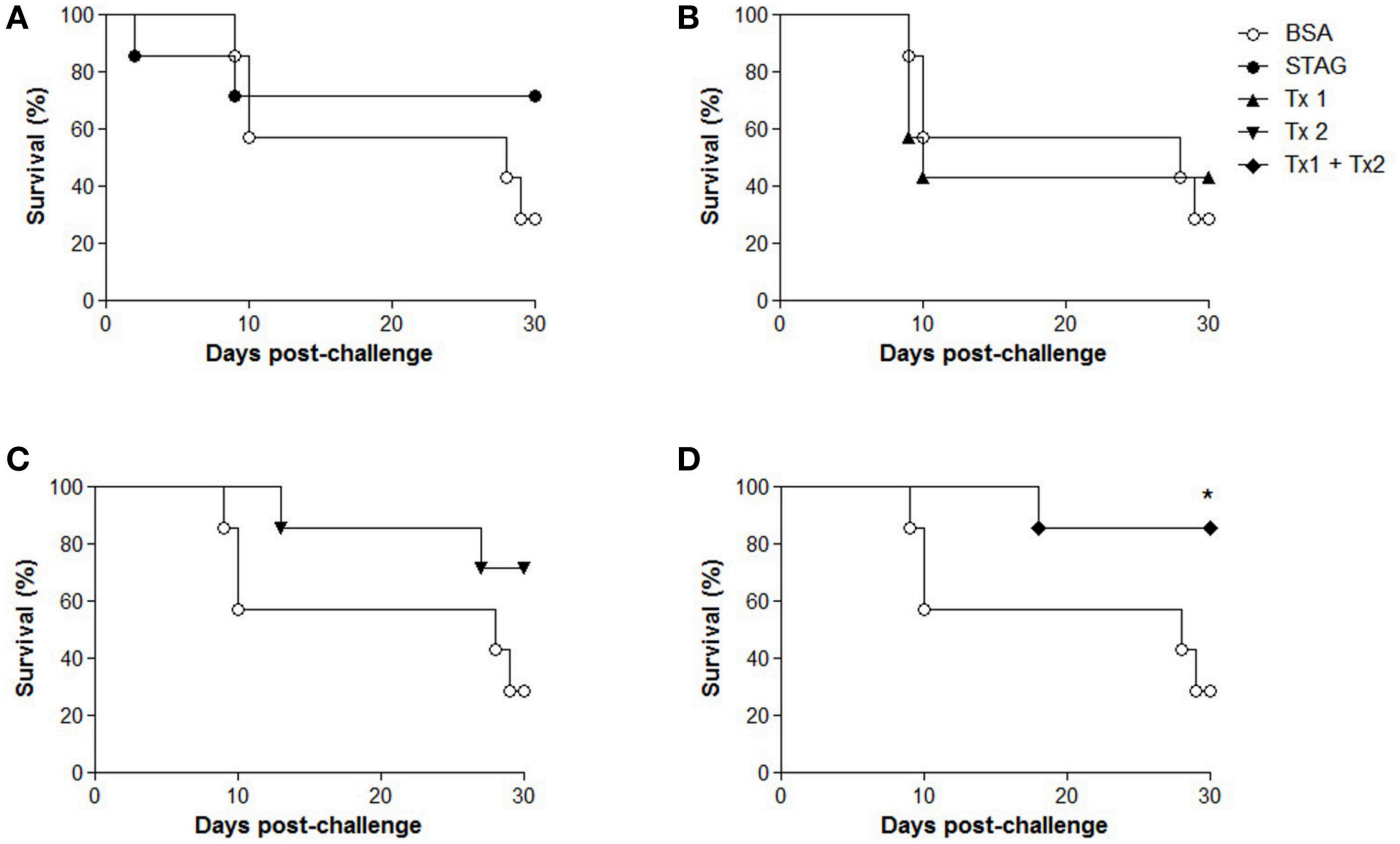
**Survival curves of C57BL/6 mice after challenge with ***Toxoplasma gondii***.** Five groups of mice (10 animals per group) were immunized with soluble tachyzoite antigen (STAg); bovine serum albumin (BSA); synthetic peptide Tx1 (Tx1); synthetic peptide Tx2 (Tx2) and both synthetic peptides (Tx1+Tx2). All curves are compared to the group immunized only with BSA. **(A)** Survival of the BSA group (28.6%) and survival group STAg (71.4%). **(B)** Survival of the BSA group (28.6%) and survival group Tx1 (42.8 %). **(C)** Survival of the BSA group (28.6%) and survival group Tx2 (71.4 %). **(D)** Survival of the BSA group (28.6%) and survival group Tx1+Tx2 (85.7%). This group showed significant difference in survival. ^*^P < 0.05.

Two weeks after the last immunization (45 d.a.i.), animals were challenged orally with 30 cysts of *T. gondii* tachyzoites and were monitored for 30 days for the evaluation of mortality. After 30 days of challenge, the higher survival rates were found for the group immunized with Tx1+Tx2 (85.7%), when compared with BSA group (28.6%; *P* < 0.05; Figure 4). To present the best protection profile of animal challenged with *T. gondii*, the next experimental conditions were carried out only with the group of animals immunized with Tx1+Tx2.

### Humoral immune response after immunization

**Figure 5:**
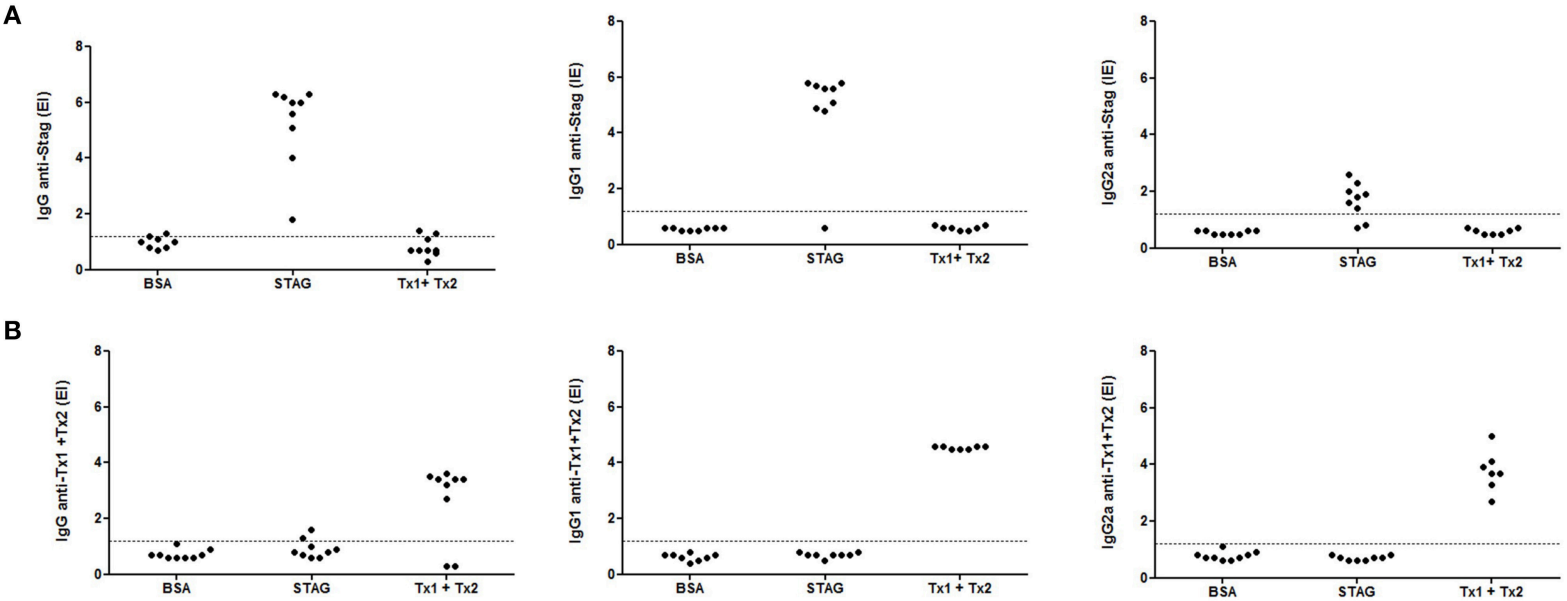
**Levels of IgG, IgG1, and IgG2a anti-STAg (A) or anti-Tx1+Tx2 (B) determined by ELISA in serum samples from C57BL/6 mice immunized with STAg, BSA or Tx1+Tx2**. It was observed that animals immunized with STAg recognized only STAg and animals immunized with Tx1+Tx2 only recognized Tx1+Tx2.

The profile IgG synthesis and its subclasses anti-STAg and anti-Tx1+Tx2 production was evaluated by an indirect ELISA. As shown in Figures 5A,B, high titers of antibodies were detected in immunized animals. Indeed, animals immunized with STAg or Tx1+Tx2 recognized only STAg or Tx1+Tx2, respectively. Higher specific IgG1 levels for STAg were observed when compared to IgG2a profile (Figure 5A). Similar recognition profile leading to the synthesis of IgG1 and IgG2a subclasses were found for the animals immunized with Tx1+Tx2 (Figure 5B).

### Cytokine production after immunization and challenge

**Figure 6:**
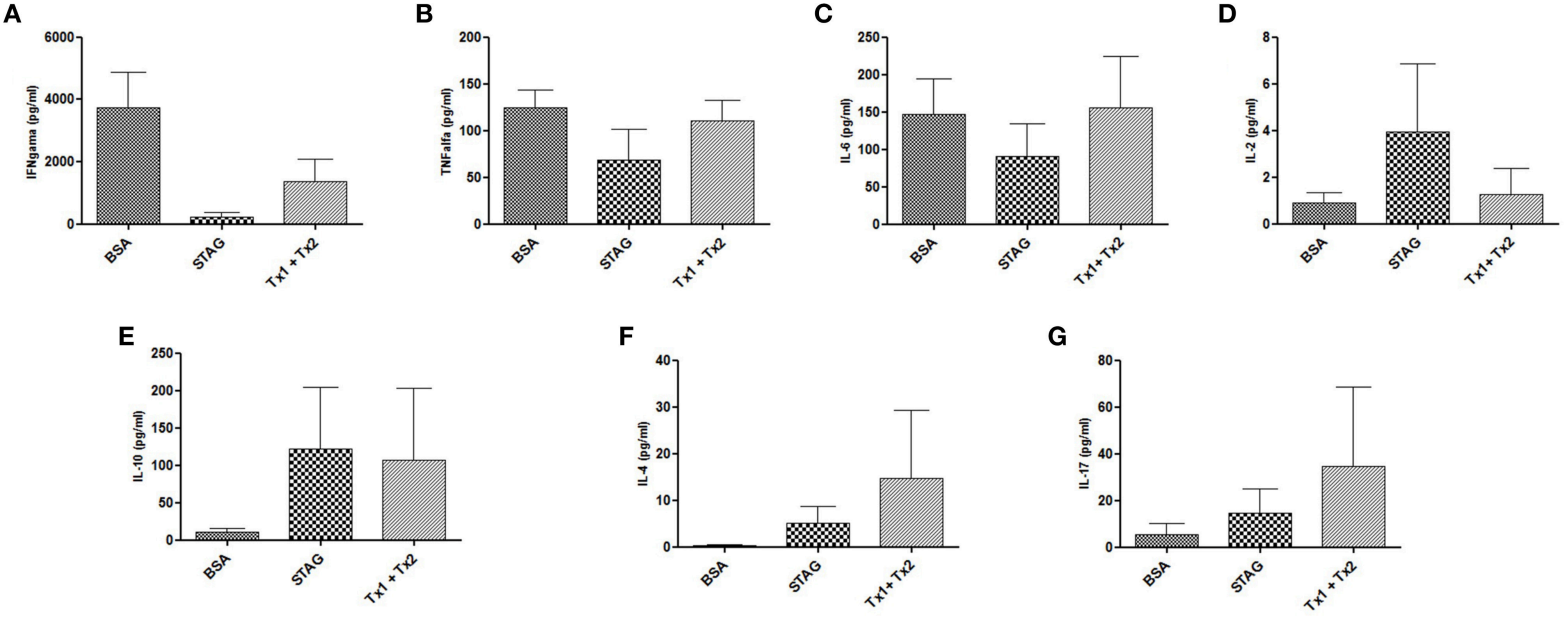
**Cytokine production by mice immunized with STAg, BSA, or Tx1+Tx2 after challenge with ***T. gondii***. (A–D)** Pro-inflammatory cytokine profiles. **(E,F)** Anti-inflammatory cytokine profile. **(G)** IL-17 production. The group immunized with Tx1+Tx2 presented a better balance between pro- and anti-inflammatory cytokines.

Cytokine production was assessed in serum samples collected 7 days after infection from animals immunized and challenged. High levels of IFN-γ, TNF-α, and IL6, in addition with very low levels of IL-10 and IL-4 were found in BSA immunized group of animals (Figures 6A–C,E,F), characterizing a predominant profile of pro-inflammatory cytokine secretion, due to the low production of anti-inflammatory cytokines for this control group of animals. In contrast, the group of animals immunized with Tx1+Tx2, as well as with STAg, demonstrated a balance between pro- and anti-inflammatory cytokines, characterized by the detection of higher levels of IL-10, IL-4, when compared with control animals, in addition with detectable levels of IFN-γ, TNF-α, and IL6 (Figures 6A–C,E,F). Also, the challenged animals from the experimental groups immunized with STAg or Tx1+Tx2 antigen preparations showed higher levels of IL-2 and IL-17, respectively (Figures 6D,G).

### Parasite burden in the brains of the immunized and challenged animals

Brain parasite burden after challenge was determined by real-time PCR. As shown in Table 1, no significant differences it was observed in the cerebral parasitism among the groups of animals immunized with BSA, Tx1, Tx2, or Tx1+Tx2, though lower levels of parasitism were found in the group of animals immunized with STAg. These findings were confirmed by direct cyst count by optical microscopy in the brains of survived animals.

**Table 1:** **Parasite burden in the brains of the immunized and challenged animals as determined by real-time PCR and by direct cyst count by optical microscopy in the brains of survived animals**.

** 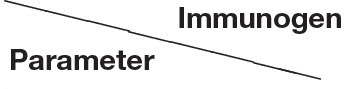 **	**BSA**	**Tx1**	**Tx2**	**Tx1+Tx2**	**STAg**
*T. gondii* DNA (ng)/brain DNA (100 ng)	0.12 ± 0.04	0.14 ± 0.05	0.18 ± 0.03	0.16 ± 0.05	0.06 ± 0.03
Number of cysts/brain	1617 ± 483	1366 ± 357	1916 ± 378	1475 ± 536	471 ± 353

*Cerebral parasitism was evaluated among the groups of C57BL/6 mice immunized with BSA, Tx1, Tx2, or Tx1+Tx2 and STAg*.

## Discussion

In the present study, we produced a monoclonal antibody to GRA2 (C3C5), a granule dense protein of *T. gondii*. Our data indicate that the C3C5 mAb recognizes a conformational epitope within the GRA2 protein, since it was recognized under SDS and heat conditions, but not under more drastic conditions, when adding a reducing agent during SDS-PAGE running. The theoretical molecular weight of GRA2 protein is 28 kDa, however, it has been described that a common feature of the dense granule proteins is the observed difference between their theoretical molecular weights and the molecular weights of the native protein estimated by SDS-PAGE analysis from lysates tachyzoites (Sibley et al., 1995; Mercier et al., 1998a). In fact, these differences may be due to post-translational modifications, such as glycosylation and phosphorylation. Furthermore, the molecular weight was assumed to electrophoresis in denaturing conditions and not reduced, so the protein structure is intact and therefore less migratory. The results of immunofluorescence assays performed with tachyzoites incubated with C3C5 mAb showed the presence of the protein mainly when the parasite membrane had been permeabilized, confirming the location of GRA2 also in the Inner Membrane Complex (IMC) of the tachyzoites, as previously demonstrated (Sibley et al., 1995; Mercier et al., 1998a). In the present study, it was also observed that the proliferation of *T. gondii* is inhibited, when the parasites were incubated with C3C5 mAb before infection of HeLa cells. Accordingly, it was already demonstrated the importance of GRA2 in the proliferation of the parasite, since this protein is involved in the maturation of the parasitophorous vacuole (PV), where the parasite replicates (Mercier et al., 2005).

In the present study, it was carried out the phage display approach to determine the binding between parasite epitope and C3C5 mAb, through the selection process of high affinity peptides binding to antibodies, named mimotopes, as previously described (Germaschewski and Murray, 1995; Smith and Petrenko, 1997; Wang and Yu, 2004). This approach has been used to determine mimotopes used to design vaccines and immunoassays to various parasitic diseases, such as malaria (Greenwood et al., 1991; Demangel et al., 1996), Chagas disease (Pitcovsky et al., 2001) and toxoplasmosis (Berghetto et al., 2003; Cunha-Júnior et al., 2010). Here, this tool has determined several mimotopes and, consequently, found that the protein was GRA2. From this, the mAb-linker peptide and another peptide selected by bioinformatics were used to proceed the immunization protocols in the animals. Our results showed that the peptide synthesized as B cell epitope did not improve survival of the immunized animals. Similar results were obtained for those animals immunized with peptide synthesized as T cell epitope, which did not increase survival significantly. In contrast, when the immunization process was carried out with both B and T cells epitopes, it was observed a survival rate higher than 85%, demonstrating that the stimulation of both cell populations induced an immune response more protective. In fact, although there was no significant difference in terms of IgG production to justify this increased survival, the immunization with both B and T cells epitopes induced a regulatory response, which was expressed by higher levels of high IL-10 and IL-4 production, whereas the group of animals immunized with BSA presented an increased inflammatory response, expressed by high IFN-γ production. It is already known that a protective immune response induced to control *T. gondii* infection requires a mixed Th1/Th2 response, with Th1 predominance (Neyer et al., 1997; Kumar et al., 1998; Meira et al., 2014). Based in our results, it can be hypothesized that the higher protection found for the animals immunized with Tx1+Tx2 can be explained by the induction of a protective immune response, expressed by a balance between stimulatory and regulatory cytokine production. It is well established in the literature that antibody response by itself is not thought to be critical during a primary infection with *T. gondii*, even though the immunoglobulin synthesis plays a significant role during the secondary immune response (Innes et al., 2009). Stimulation of the innate immune system occurs early in *T. gondii* infection, because the parasites are able to stimulate macrophages directly resulting in the production of IL-12, which in turn can stimulate NK cells to produce IFN-γ (Gazzinelli et al., 1993; Innes et al., 2009). This early induction of IFN-γ is important to inhibit tachyzoite proliferation during the early stages of infection by providing the appropriate cytokine environment during the priming of the adaptive immune response, which results in a bias toward a Th1-type pro-inflammatory immune response (Gazzinelli et al., 1996; Innes et al., 2009). The regulatory cytokine IL-10 is essential for protection against the potential immunopathological mechanism caused by a vigorous Th1-type immune response (Gazzinelli et al., 1996; Innes et al., 2009).

When using immunocompetent animals, *T. gondii* experimental infection results in development of protective immunity against toxoplasmosis, since the challenge has been carried out using strains from the same genotype. It is already well documented in the literature that there are significant differences in virulence and immune response between the different genetic types of *T. gondii*. This phenomenon has been described among clonal genotypes, as well as among those called atypical/recombinant strains, as it has been observed by our group also (Franco et al., 2015; Lopes et al., 2016). Concerning GRA2 molecule, even though there are 98.4% identity and 98.9% similarity between type I and type II *T. gondii* genotypes, three polymorphisms have been identified, which are located at 95, 166, and 167 AA positions. In the present study, it was design the Tx1 and Tx2 predicted peptides, which are located in a 100% conserved region of GRA2 molecule from RH and Me49 *T. gondii* genotypes, at 68–76 AA positions. The main reason to carry out our experiment design, i.e., using a type I strain (RH) to produce peptide and then challenged immunized mice with the Me49 type II strain, was because we were interested to observe both survival rates and parasite burden, expressed by formation of brain cysts. Thus, it was not possible to challenge the mice with RH strain, due to the fact that this strain does not form brain cysts in C57BL/6 mouse genotype, which would die very rapidly, during the acute phase of the infection, after 7–10 days.

To control toxoplasmosis, the vaccination process has a high likelihood of success, but the selection of an appropriate antigen preparation constitutes the major challenge. There is a commercial vaccine available to protect against *Toxoplasma* abortion in sheep and goats, named Ovilis1 Toxovax (Intervet) (Innes and Vermeulen, 2006). This vaccine contains attenuated live tachyzoites from the S48 strain, which does not produce oocysts, but it is unable to prevent vertical transmission (O'Connel et al., 1988). This vaccine has been licensed for veterinary use only, because there is no data available so far, concerning its safety to be used in humans. Therefore, studies leading to the development of a human vaccine able prevent congenital toxoplasmosis have focused in using defined immunodominant antigens and different delivery strategies (Innes and Vermeulen, 2006). It has been shown that CD4^+^ T cell *Toxoplasma*-specific clones from chronically infected healthy donors react with both SAG1 and GRA2, supporting the hypothesis that a combination of these antigens or appropriate derivative peptides represents suitable candidates for vaccine development in humans (Prigione et al., 2000). In this context, stimulation of immune response toward GRA2 peptides may be important to control infection, as immunization with the native protein has been shown to protect mice against acute toxoplasmosis (Sharma et al., 1984; Mercier et al., 1998b).

In conclusion, our results demonstrate that the immunization protocol using synthetic epitopes from *T. gondii* GRA2 protein is able to stimulate both B- and T-cells and can be effective to protect against the parasite infection, corroborating with the findings regarding the immunogenicity of GRA2 and confirming that peptides carrying these features from this protein may be useful candidates to be taken into account in vaccination design protocols.

## Authors contributions

LB was involved in monoclonal antibody preparation, selection of epitopes by phage display, mouse immunization procedures and parasite challenge, cytokine and antibody assays, statistical analysis, determination of brain parasite load for qPCR and preparation of the draft manuscript. AM participated in proliferation assay, mouse immunization procedures and parasite challenge, cytokine and antibody assays, statistical analysis and preparation of the draft manuscript. MS was involved mouse immunization procedures and parasite challenge, cytokine and antibody assays and statistical analysis. FS was involved in the parasite maintenance in cell culture, antigen preparation, and monoclonal antibody preparation. ER participated in Indirect fluorescent antibody test. CP was responsible for the sequencing experiments. FS, LG participated in the selection of epitopes by phage display. TM, JM were involved in the experimental design, bioinformatics procedures, data analysis and revision of the manuscript. All authors read and approved the manuscript.

## Funding

This work was supported by Brazilian Research Agencies - CNPq (Proc.# 311787/2013-4 and 456650/2013-0), FAPEMIG (Proc. #APQ-01313-14), and CAPES (Proc. #AUXPE-02450/09-7).

### Conflict of interest statement

The authors declare that the research was conducted in the absence of any commercial or financial relationships that could be construed as a potential conflict of interest.

## Abbreviations

GRA: dense granules proteins
PV: parasitophorous vacuole
STAg: soluble tachyzoite antigen
ME: mercaptoethanol.
